# Metabolic footprinting of extracellular metabolites of brain endothelium infected with *Neospora caninum* in vitro

**DOI:** 10.1186/1756-0500-7-406

**Published:** 2014-06-28

**Authors:** Hany M Elsheikha, Mamdowh Alkurashi, Kenny Kong, Xing-Quan Zhu

**Affiliations:** 1 School of Veterinary Medicine and Science, Faculty of Medicine and Health Sciences, University of Nottingham, Sutton Bonington Campus, Leicestershire LE12 5RD, UK; 2 Animal Production Department, College of Food and Agricultural Sciences, King Saud University, Riyadh 11451, Saudi Arabia; 3 School of Physics and Astronomy, University of Nottingham, Nottinghamshire NG7 2RD, UK; 4 State Key Laboratory of Veterinary Etiological Biology, Key Laboratory of Veterinary Parasitology of Gansu Province, Lanzhou Veterinary Research Institute, Chinese Academy of Agricultural Sciences, Lanzhou, Gansu Province PR 730046, China

**Keywords:** Adaptation, Blood brain barrier, Host-pathogen interaction, Metabolic footprinting, Metabolomics, *Neospora caninum*

## Abstract

**Background:**

The survival of the intracellular protozoan parasite *Neospora caninum* depends on its ability to adapt to changing metabolic conditions of the host cell. Thus, defining cellular and metabolic changes in affected target tissues may aid in delineating pathogenetic mechanism. We undertook this study to assess the metabolic response of human brain microvascular endothelial cells (HBMECs) to *N. caninum* infection *in vitro*.

**Methods:**

HBMECs were exposed to *N. caninum* infection and the cytotoxic effects of infection were analyzed by the 3-(4,5-dimethyl-2-thiazolyl)-2,5-diphenyl-2H-tetrazoliumbromidin (MTT) assay and lactate dehydrogenase (LDH) release assay. Metabolic footprinting of the extracellular metabolites of parasite-infected and non-infected culture supernatant was determined by using targeted (Randox RX Imola clinical chemistry analyser) and unbiased RS (Raman microspectroscopy) approaches.

**Results:**

The MTT assay did not reveal any cytotoxic effect of *N. caninum* challenge on host cell viability. Measurement of LDH activity showed that *N. caninum* significantly induced loss of cell membrane integrity in a time-dependent and dose-dependent manner compared to control cells. Targeted biochemical analysis revealed that beta hydroxybutyrate, pyruvate, ATP, total protein, non-esterified fatty acids, and triglycerides are significantly different in infected cells compared to controls. RS-based footprinting with principal component analysis (PCA) were able to correctly distinguish extracellular metabolites obtained from infected and control cultures, and revealed infection-related spectral signatures at 865 cm^−1^, 984 cm^−1^, 1046 cm^−1^, and 1420 cm^−1^, which are attributed to variations in the content of lipids and nucleic acids in infected cultures.

**Conclusions:**

The changing pattern of extracellular metabolites suggests that HBMECs are target of metabolic alterations in *N. caninum* infection, which seem to reflect the changing metabolic state of infected cells and constitute a level of information exchange that host and parasite use to coordinate activities.

## Background

The complex interaction between intracellular (IC) pathogens and their eukaryotic host cells embodies the fundamental evolutionary struggle of eukaryotic cells to survive under a continuous challenge caused by the infecting pathogen. Despite significant progress in the past decades, how an obligatory IC apicomplexan protozoan parasite, such as Neospora caninum adapts to host cell microenvironment, and the implication of this on the viability of the host cell and the fitness of the parasite remains largely unknown. This parasite infects a large number of vertebrate animals and is responsible for abortion and infertility problems in cattle and neuromuscular disease in dogs [1,2]. However, N. caninum infection is generally latent and asymptomatic, and results in the formation of dormant cysts that remain in the brain and other tissues for life [1]. As an obligate IC pathogen, N. caninum survival is dependent upon entry, growth and development within the eukaryotic host cell and then exiting to initiate a new infection cycle. The IC infection cycle ends up with lysis of the host cell and release of the parasite progeny. Despite significant research efforts understanding of the cellular processes by which N. caninum initiates infection and cause disease remains incomplete, partly, due to the complexity of N. caninum-host interaction, which determines the host response and outcome of infection.

Critical aspects of N. caninum infection occur in CNS tissues, particularly at the blood brain barrier (BBB) interface. N. caninum is a neuro-pathogen with a remarkable capacity to cross the BBB and infect neurons and other brain cells, with adverse consequences [3,1]. However, many aspects of the molecular basis of neuropathogenicity of N. caninum have not been fully elucidated. For example, our knowledge about the substrates used by N. caninum during infection, and the effect of N. caninum infection on the metabolism of the host cell is unknown. Giving the significant animal health implication and economic losses associated with N. caninum infection better understanding of the biochemical and metabolic changes in BBB cells induced by N. caninum and the metabolic requirement of N. caninum during infection is essential in order to understand the parasite neuro-pathogenesis. Recently, we investigated changes in BBB endothelial cell bioenergetics in response to N. caninum infection [4]. It is important to examine what makes the BBB endothelial cells a tolerant environment for the growth of N. caninum.

N. caninum is largely dependent on the host cell to obtain the nutrients that are necessary for its replication. N. caninum-infected cells are expected to have different metabolic requirements from their normal (uninfected) counterparts because replication of IC parasites requires energy for synthesis of macromolecules, such as proteins, DNA, lipids, which are essential for the assembly of the growing parasites. Hence, N. caninum-infected cell faces two major metabolic challenges: (1) how to meet the bioenergetic and biosynthetic demands of the growing IC parasites and (2) how to adapt to the fluctuations in the extracellular nutrient availability. Understanding the consequences of this differential metabolic stress requires a detailed understanding of host cell metabolism and viability in infected cells compared to healthy control cells. Metabolomic technologies can provide a global analysis of cellular phenotype in response to infection. However, methods used for sampling intracellular metabolites without changing their relative concentrations or introducing contamination from supernatant metabolites is not optimal [5,6]. In contrast, exometabolome or metabolic footprinting is simple, and extracellular metabolites can exhibit very large changes in pool size. Exometabolome analysis has been used for phenotyping of microbial populations [7-10] or studying cellular response to drugs [11].

In the present study we investigated the hypothesis that pathophysiological effect of *N. caninum* infection on host tissue can be attributed to changes in the metabolic status of host cells during the course of infection. Our goals in this study were to: (i) seek direct evidence for the alteration in the metabolic response of brain microvascular endothelial cells, a fundamental component of the BBB, to *N. caninum* infection and (ii) determine the level and kinetic of extracellular metabolites in culture medium from *N. caninum*-infected versus control cells.

## Methods

### Ethical consideration

This study was reviewed by the University of Nottingham (UK) School of Veterinary Medicine and Science (SVMS) Ethical Review Committee. The Committee reviews all research studies involving School personnel and is chaired by Professor David Haig. The committee passed this in vitro study as good to proceed, not requiring any further ethical review as it doesn’t involve vertebrate or invertebrate animals.

### Cell line

Human brain microvascular endothelial cells (HBMECs) were maintained as described previously [4], in complete RPMI-1640 (cRPMI) medium supplemented with 20% heat inactivated fetal calf serum (FBS), 2 mM L-glutamine, 1 mM Sodium Pyruvate, 1 mM MEM non-essential amino acids, 1% MEM vitamins and 100 units/mL penicillin/streptomycin at 37°C under humidified 5% CO_2_ conditions. When cells were confluent they were harvested with trypsin-EDTA and passaged at a sub-cultivation ratio of 1:3 into new culture flasks with fresh medium. Cells were considered confluent when their expansion had reached a point where cells touched each other on all sides, leaving no intercellular spaces. To exclude if cell viability could be regarded as a factor affecting response of the host cell to parasite infection and hence any subsequent metabolic analysis, the number of viable cells was determined on a minimum of 100 cells by hemocytometer under a light microscope after staining with 0.15% trypan blue solution. Cells used in the experiments had a viability not less than 99% at all times.

### Parasites culture

Neospora caninum (Nc-Liverpool) strain was propagated in Vero cells as described [12]. Infected host cell monolayers were scraped, parasites were isolated from host cells by passage through 25- and 27-gauge needles and purified by using PD-10 Desalting Columns prepacked with Sephadex G-25 medium as described previously [13]. Purified parasites were centrifuged at 800 × g, washed twice with fresh cRPMI, re-suspended in fresh medium and quantified using a hemacytometer. The final volume of suspension was adjusted with cRPMI medium to achieve a ratio of 2:1 parasite/host cell for subsequent infection experiments. Parasite viability was checked by using trypan blue staining assay and parasite with more than 97% viability were used.

### *In vitro* infection protocol

Cells (3 × 10^5^ cells/mL) were seeded at the bottom of 6-well culture plates with a volume of 2 mL cRPMI medium/well. Cells were allowed to grow overnight by incubation at 37°C in a humidified atmosphere with 5% CO_2_ in air. Before infection, cell growth medium was removed and cells were washed three times with sterile PBS (8 g/L NaCl, 0.2 g/L KCl, 0.2 g/L KH_2_PO_4_, 1.15 g/L Na_2_HPO_4_). Then, in each 6-well plate, three wells were infected with parasites at a MOI of 2 in 2-ml fresh medium, and the remaining three wells received only 2-ml fresh medium (mock-infected) and considered controls. Culture plates were then incubated to allow infection to progress within cells. Culture media were sampled at different time point post infection (PI) starting from 0 h, and then, at 1, 2, 3, 6, 12, 18, 24, 48 h PI. At each sampling time six wells (three infected and three controls) were collected and centrifuged at 1000 × g for 3 min, and the supernatants collected and kept at −80°C until analysis of extracellular metabolites.

### MTT assay

The nonradioactive metabolic assay MTT (3-(4,5-dimethyl-2-thiazolyl)-2,5-diphenyl-2H-tetrazoliumbromidin ) was used to assess the effect of N. caninum infection on the viability of host cells. HBME cells were trypsinized from T-75 culture flasks, seeded into 96-well tissue culture microtiter plates (Nunc) at 1 × 10^4^ cells per well in 100 μl of culture medium, and incubated for 18 h in a humidified incubator (37°C, 5% CO2) until become confluent. N. caninum tachyzoites were added to the cells at 2 MOI for 2 h, followed by removal of the medium and 2x washing with fresh medium to remove unbound parasites and cellular debris. Each well was then filled with 100 μl of fresh culture medium and plates were incubated at the above culture conditions. As a positive control, cells were treated with 1 μM staurosporine, an apoptotic agent. Cell viability was measured at 3, 6, 12, and 24 h PI by the reduction of MTT in a colorimetric assay. Briefly, MTT (Sigma Chemical, St. Louis, MO, USA) was added to each well (to a final concentration of 0.5 mg/ml), and incubation was continued for 4 h in the dark at 37°C. The cells were then incubated for 1 h in solubilization solution (50% sodium dodecyl sulfate in 0.1 mM/L HCl). The spectrophotometric absorbance of the samples was subsequently measured with a microtiter enzyme-linked immunosorbent assay (ELISA) plate reader using a 570-nm filter. The level of MTT reduction was expressed as a percentage of that of the non-infected control cells. The assay was performed in triplicate.

### Lactate dehydrogenase assay

Lactate dehydrogenase (LDH) activity released into the culture medium (a measure of cell membrane lysis due to necrotic cell death) was assayed using a CytoTox 96 Kit (Promega, Madison, Wis.) according to the manufacturer’s instructions. Briefly, 1 × 10^4^ HBMECs were seeded onto sterile 96-well plates and grown until 90% confluence and subsequently infected with N. caninum tachyzoites using different multiplicities of infection (MOIs) ranging from 0.5 to 4. After 3, 6, 9, 12, 18, and 24 h of incubation, the supernatants were collected, centrifuged to obtain cell-free supernatants. Of each sample, 50 μl per well was transferred to new 96-well plates. LDH activity was detected by the addition of freshly prepared reagents followed by incubation for 30 min in the dark at ambient temperature. LDH activity was measured by a redox reaction that couples the oxidation of lactate iodotetrazolium chloride to a colored formazan salt, using NADH as the electron transfer agent and NADH diaphorase as the catalyst. The absorbance at 490 nm was read using a Bio-Tek Instruments EL311SX plate reader. The cytotoxicity was expressed as a percentage of maximum LDH release, i.e., 100 × (optical density at 490 nm [OD490] of infected cells − OD490 of uninfected cells)/(OD490 of 2% Triton X-100-lysed uninfected cells − OD490 of uninfected cells). This assay was performed in triplicate wells, and the data represent the mean ± standard error of the mean (SEM) from at least three separate experiments. Statistical analysis was calculated by Student’s t-test using the Graph Pad Prism 3.0 statistical program (GraphPad Software Inc., San Diego, CA). P < 0.05 was taken to indicate statistical significance.

### Biochemical analysis of extracellular metabolites

The level of the 20 metabolites was determined colorimetrically in culture medium obtained from infected and control wells at different time points PI using commercially available kits and a Randox RX Imola clinical chemistry analyzer (Randox Laboratories Ltd., Belfast, UK) according to the manufacturer’s specifications. Biochemical parameters measured included albumin (AB3800), glucose hexokinase (GL3816), calcium (CA3871), magnesium (MG3880), phosphorus (PH3820), NEFA (FA115), BHB (RB1007), cholesterol (CH3810), TGA (TR3823), total protein (TP3869), urea (UR3825), lactate (LC3980), chloride (CL1645), sodium (NA3851), potassium (PT3852), iron (SI3821), HDL (CH3811), and LDL (CH3841). Additionally, pyruvate and ATP were measured. All reagents used in the experiment were of analytical grade, or better. All of the 20 metabolites were quantified at each sampling time in order to assess the fluctuation in their concentration in culture medium in response to the progression of infection within cells. In all parameters, the data are the means of at least three independent experiments ± SEM. Statistical difference in metabolite concentrations were examined by ANOVA for repeated measurements (Genstat 12, VSN International, Hemstead, UK). The main effect tested was exposure to infection. A one way ANOVA was used to determine significant differences between control and infected cell cultures over a range of pre-determined periods of time PI. Probability values *P* < 0.05 were considered to be significant.

### Raman micro-spectroscopic analysis

The level of the culture metabolites was determined by Raman microspectroscopy imaging. Raman spectra were recorded using a custom built Raman micro-spectrometer based on an inverted optical microscope (Eclipse-Ti, Nikon) with a Leica 50×/0.55 objective, 785 nm wavelength laser (Starbright XM, Torsana), spectrometer (77200, Oriel), back-illuminated deep-depletion CCD (DU401-A-BR-DD, Andor Technology) and automated sample stage (H107 Proscan II, Prior Scientific). The acquisition time for the Raman measurements was 1 second per position and the laser power on the sample surfaces was 200 mW. The spectrometer was calibrated using naphthalene and 1,4-bis(2-methylstyryl) benzene samples (both from Sigma-Aldrich, UK) to an accuracy of 0.5 cm^−1^. From each sample of culture medium a drop of ~50 μl was placed on a MgF2 coverslip fitted in a titanium chamber. About 225 Raman spectra were uniformly measured over 50 × 50 μm^2^ area. These were then averaged, and Raman spectra were subtracted from ‘control’. Spectra from all samples were analysed using Matlab R2013a software. The experiment was conducted in triplicates and was repeated twice. Raman spectra were assigned by comparison of their chemical shift to published values. Due to the qualitative similarity and complexity of the spectra from the infected and control cultures, a visible inspection of differences would be very difficult and so an independent multivariate analysis was carried out with PCA, to investigate basic biological differences between the medium of infected versus non-infected cell cultures.

## Results

### Cell viability

**Figure 1:**
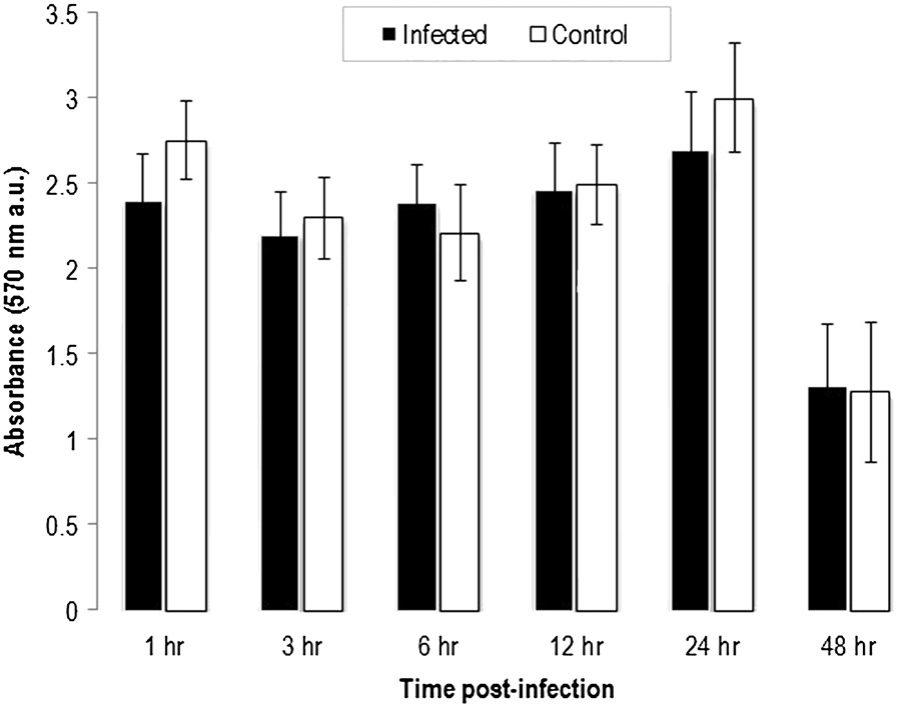
**Metabolic activity of non-infected and *Neospora caninum-*infected HBMECs.** Effect of *N. caninum* infection on the cell viability of HBMECs was determined by an MTT assay at the indicated time points post infection. No significant differences were observed between infected and control cells at any of the tested time points.

**Figure 2:**
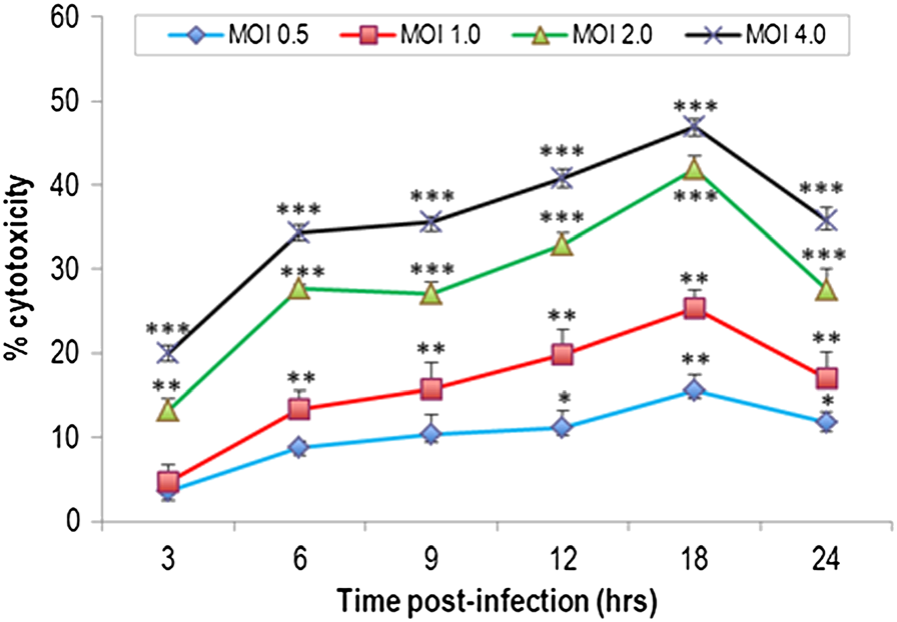
**Time course, dose–response curves for *Neospora caninum *cytotoxicity. ***N. caninum* caused cytotoxicity to endothelial cells in a dose-dependent manner. Cells were cultured in 96-well plate and were infected with *N. caninum* at different MOIs as described in methods. The supernatants were collected at the indicated time points, and were assayed for the cytosolic enzyme lactate dehydrogenase released by cells, using the CytoTox96 nonradioactive cytotoxicity assay. Data are expressed as mean ± SEM of three independent experiments performed in triplicate. *P < 0.05; **P < 0.01; ***P < 0.001 versus the control group, respectively.

The effect of N. caninum on the metabolic activity of HBMECs was assessed by using two methods. First, the MTT assay was used to assess changes in the cells’ viability in exposure to N. caninum. As shown in Figure 1 there was no significant difference between the metabolic activity of infected- and non-infected HBMECs at each time point (P > 0.05) (ANOVA statistical analysis). Secondly, to determine if N. caninum has any adverse effect on the membrane permeability of infected cells, LDH activity in supernatants of HBMECs infected with N. caninum was compared with mock-infected cells (Figure 2). For all four MOIs, an increase in cell cytotoxicity was found to increase in a dose- and time-dependent manner reaching maximum activity at 18 h PI, followed by decrease at 24 h PI. There was no statistical difference in LDH activity between MOI 0.5 and 1, or 2 and 4. However, significant statistical difference was observed between the control and all different MOIs during the whole experimental period.

### *N. caninum* triggers metabolic changes in HBMECs

**Figure 3:**
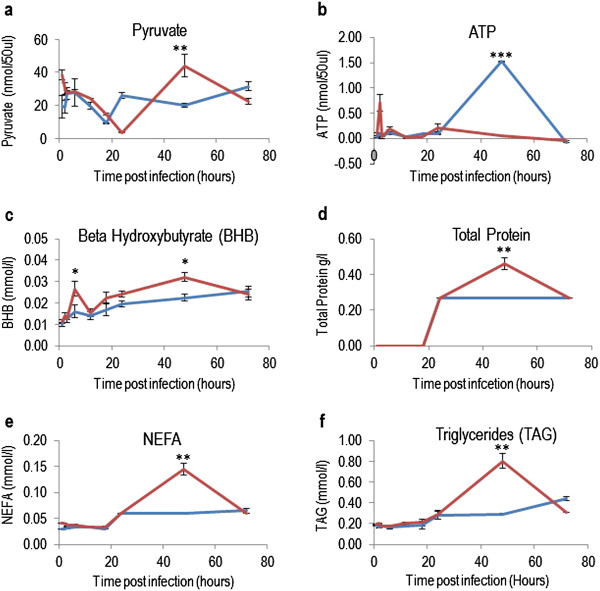
**Time-specific metabolic changes in medium of *Neospora caninum *-infected and mock-infected culture of human brain microvascular endothelial cells.** Temporal changes in the concentrations of pyruvate **(a)**, ATP **(b)**, β-hydroxybutyrate (BHB; **c**), total protein **(d)**, non-esterified fatty acids (NEFA; **e**), and triglycerides (TGA, **f**) in culture medium of infected (red line) versus control (blue line). The statistical analysis was performed for every time point separately. *P < 0.05; **P < 0.01; ***P < 0.001 versus the control group, respectively. Error bars indicate the standard errors of the means (*n* = 6).

Dynamic changes in the concentration of 20 biochemical parameters were determined in the culture media of infected and control cultures. The following 14 metabolites did not show statistically significant difference between infected and control cultures: phosphorus, magnesium, calcium, chloride, sodium, potassium, iron, lactate, HDL, LDL, glucose hexokinase, urea, albumin, and cholesterol. On the other hand, the levels of pyruvate, ATP, BHB, total protein, NEFA, and TGA were statistically different between infected and control cell cultures at certain time points PI (Figure 3). Pyruvate level was significantly higher in infected culture compared to control at 48 h PI. ATP level in the culture supernatant changed during the parasite growth (Figure 3). During the first 24 h PI there was no difference in the level of ATP between infected and control culture, followed by increased consumption (i.e. reduced extracellular concentration) of ATP in *N. caninum*-infected cells till the end of the experiment (72 h PI), corresponding with the increase in the parasite growth within host cells. BHB levels were elevated in infected culture from 3 to 48 h PI, and this elevation was significantly higher (p < 0.05) in infected culture compared to control at 6 h PI and between 12 and 48 h PI (Figure 3). A reduction in the levels of total protein, NEFA, and TGA were observed in control culture media from 24 to 72 h PI.

### Raman spectra of culture media

**Figure 4:**
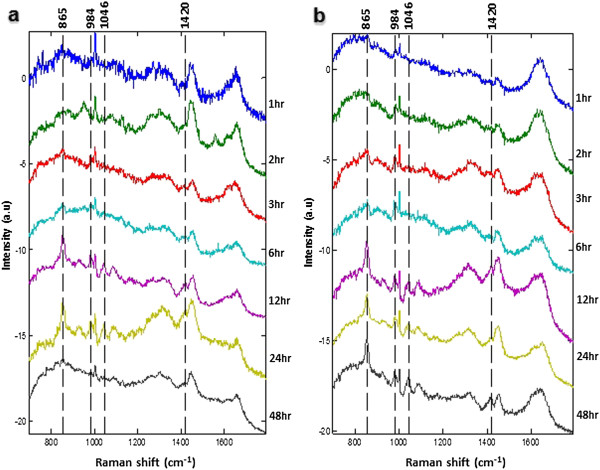
**Comparative time-resolved metabolic footprinting of extracellular metabolites using Raman microspectroscopy.** Average Raman spectra of medium of **(panel b)** noninfected- and **(panel b)***Neospora caninum*-infected HBMECs at different time points post infection. Dotted lines indicate the position of the Raman bands at 865 cm^−1^, 984 cm^−1^, 1046 cm^−1^ and 1420 cm^−1^.

**Figure 5:**
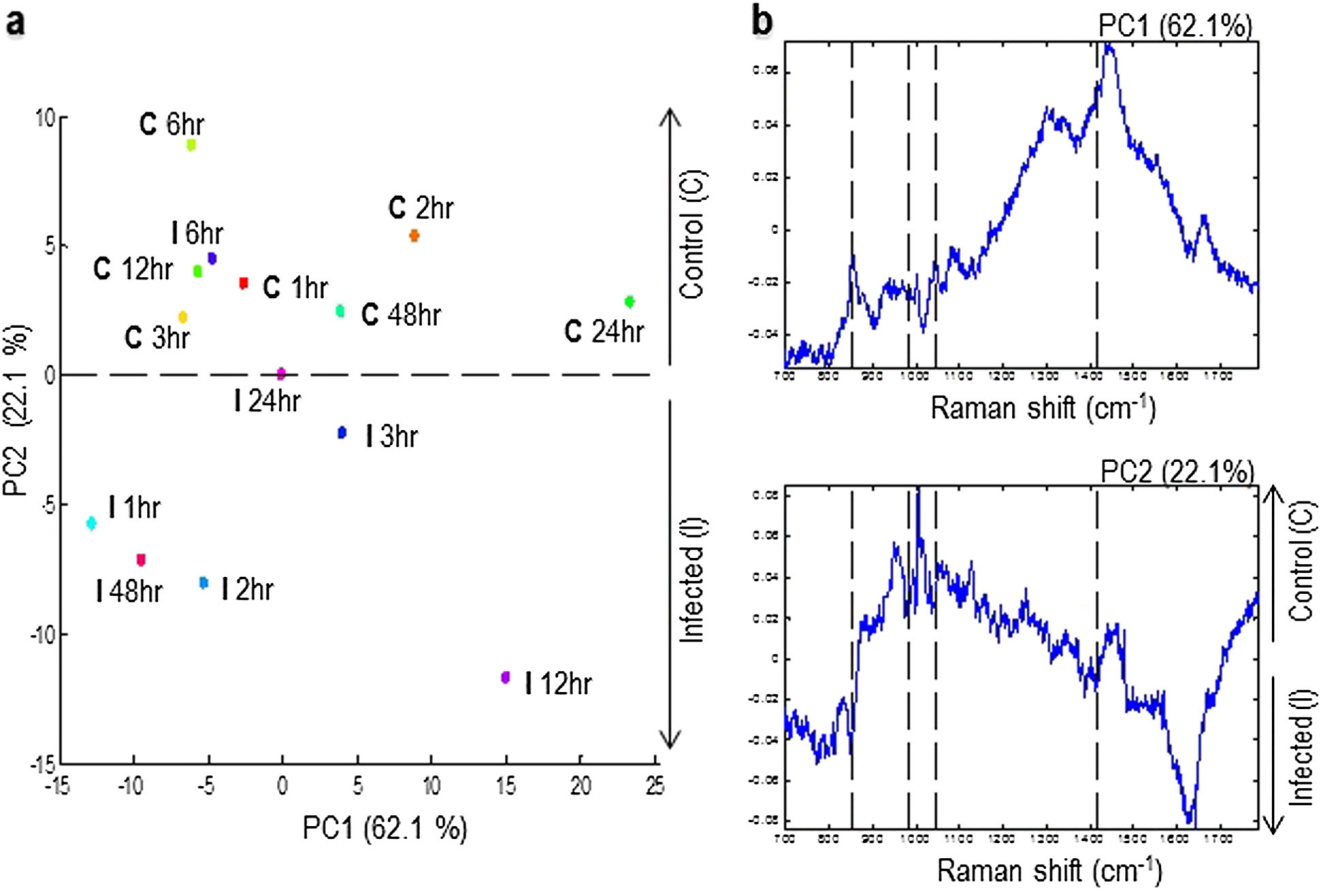
**PCA discrimination of medium from healthy and *Neospora caninum*-infected cell culture media with respect to time post infection. Panel a** depicts the PCA for time-resolved Raman spectral data, score plot of axes 1 and 2. Principal components 1 and 2 explained 62.1and 22.1% of the variance in the data, respectively. Samples at different time points are indicated by different colours. **Panel b** depicts the loadings plot for the analysis shown in panel A showing the discrimination between the spectra of control (C) and (I) infected culture media. The dotted lines in loading plots mark the location of the Raman bands depicted in Figure 4.

**Figure 6:**
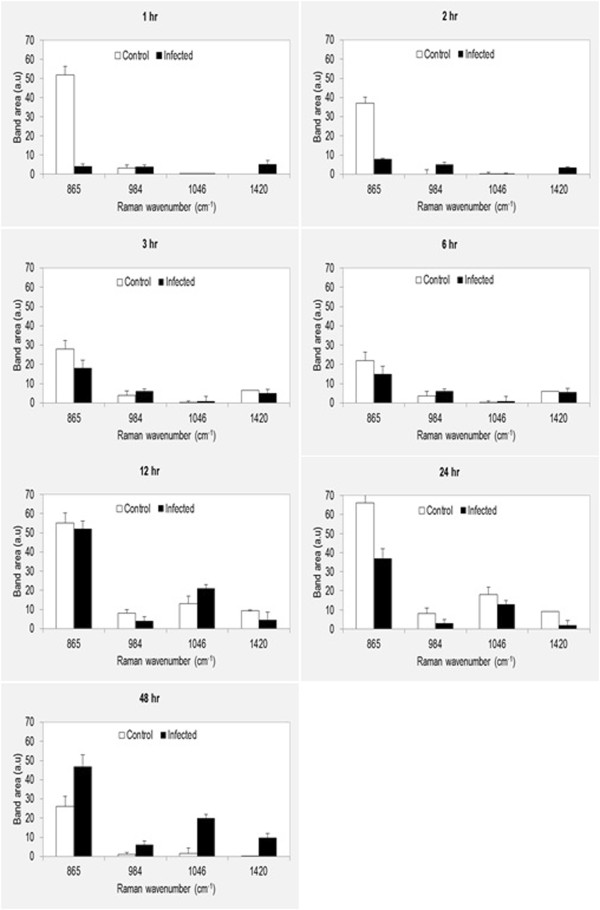
**Time-resolved intensities of Raman peaks comparing the spectra of culture media from normal and infected cultures over time points post infection.** The main differences are in the intensity variations. At the late stage of the intracellular life cycle of the parasite (48 h), peaks at 865 cm^−1^, 984 cm^−1^, 1046 cm^−1^, and 1420 cm^−1^ are more intense in the infected culture than in the control culture.

Raman spectral patterns of medium from non-infected and infected cultures were obtained at different time points PI. Label-free imaging analysis was able to detect variations in the biochemistry of culture media between infected and control cell cultures and to reveal spectral signatures of even low-concentration metabolites. As shown in Figure 4 comparative Raman spectral analysis revealed many similarities and few differences, with prominent peaks at 865 cm^−1^, 984 cm^−1^, 1046 cm^−1^ and 1420 cm^−1^. These spectra are arising from contributions of lipids (865 cm^−1^ and 984 cm^−1^) and nucleic acids (1046 cm^−1^ and 1420 cm^−1^). Independent component analysis, PC3 and PC4 were not able to distinguish between metabolites from infected and control culture media. However, PC1 and PC2 enabled the separation between the spectra from infected and control cultures (Figure 5). These loadings plots indicate that PC1 and PC2 account for the greatest variation within the Raman dataset and revealed an infection-related trend. Trends according to spectra from time points showed a pattern of Raman signals 865↑, 984↑, 1046↑, and 1420↑ for infected medium at 48 h PI (Figure 6). The higher DNA and lipid contents in infected cultures seem to reflect the added contribution of DNA and membrane lipid from the growing parasites.

## Discussion

The high metabolic demands needed for the growth of IC parasites are expected to trigger different physiological responses in host cells. For cells to handle such a challenge they have to adapt their metabolism to compensate for the bioenergetic needs of the growing parasites. N. caninum was able to invade and replicate within HBMECs without altering the metabolic activity at any time point up to 48 h PI as assessed by the MTT assay (Figure 1), which is in agreement with results from our previous study [4]. However, N. caninum infection was correlated with the induction of dose- and time-dependent cytotoxicity in HBME cells as assayed by LDH release (Figure 2).

We monitored changes in several metabolites in cell culture supernatant using targeted biochemical assays and, as illustrated in Figure 3, significant changes in key metabolites have been detected in culture media in response to infection. ATP is the primary energy currency of living organisms and participates in a variety of cellular processes. The reduced ATP level in the medium of infected cultures is perhaps due to the depletion of glucose caused by increased energy consumption by the infected cells. Alternatively, reduced ATP might be attributed to parasite salvage of host cytosolic ATP, which affects the host cell’s metabolism leading to alterations in the ATP concentration in the medium. In agreement with our finding, a recent study reported an association between low level of purine and early phase of experimental N. caninum infection in gerbils [14].

Infection seems to cause a shift in the main energy producing pathway (trichloroacetic acid cycle) towards increased mobilization of ketone bodies especially BHB. Interestingly, HBMECs seem to be capable of compensating for glucose restriction by metabolizing ketone bodies *in vitro*, suggesting a potential advantage of infected host cells. Glucose is a fundamental nutrient required for normal function of brain and other tissues. It is under tight homeostatic control to allow for basic functioning of the host organism. In response to a limited glucose, an increase in lipolysis releases NEFA, which can be used as a fuel source by host cells. NEFA can be oxidized to provide energy and to produce ketone bodies (acetone, acetoacetic acid, and BHB), and converted into TAGs. Consistent with this hypothesis is the markedly increased levels of the total protein, NEFA and triglycerides in infected culture compared to the level measured in non-infected culture.

While a certain concentration of BHB and NEFA is a normal adaptation to limited nutritional resources excessive concentrations of NEFA or BHB indicate an excess of negative energy balance, which can be detrimental to the health of the host cells. Negative energy balance seems to occur because the energy demands for infected cells cannot be completely met due to the increasing metabolic demands of the proliferating parasites. BHB seems to influence host-pathogen interaction in different ways, impinging on cell metabolic adaptation and on its interaction with the parasite. BHB, besides providing an alternative fuel source to infected cells, prevents apoptotic and necrotic cell death [15], supporting the survivability of the parasite. BHB can also potentiate infection in different ways. For example, BHB was shown to increase vascular permeability of brain microvascular endothelial cells in a murine model [16], facilitating the parasite crossing to the brain. Also, BHB was found to negatively affect bovine neutrophils, increasing susceptibility of ketotic cows to mastitis and other infectious conditions [17]. Interestingly, lower neutrophil count has been recorded in N. caninum-seropositive cows [18]. Further experiments should confirm the biological relevance of the BHB in this parasite infection.

Lipid droplets, major lipid storage structures, are composed of a triglyceride and cholesteryl ester core with a surrounding monolayer of phospholipid, cholesterol, and a variety of associated proteins with diverse functions in cell metabolism, signaling, and inflammation [19,20]. The interaction of parasite proteins with these lipid bodies is important for the replication of IC, such as N. caninum and for the biogenesis of new parasite particles [21]. Interestingly, culture medium concentrations of TGAs and NEFAs significantly increased by N. caninum infection, which is consistent with a previous report where the size and number of lipid droplets whose major component is TGAs increased in cells infected by a related protozoan, Toxoplasma gondii [21]. The endogenously generated unsaturated FAs may play a role in TGA accumulation, and it is possible that unsaturated FAs activate signaling pathways that promote TGA storage (or inhibit TGA hydrolysis). Although infection increases cellular neutral lipids, including TGAs, the cholesterol contents did not seem to be significantly affected by N. caninum infection.

The marked increase in the concentrations of TGAs and NEFA in infected culture medium compared to control and the significant changes in expression of genes involved in lipid biogenesis (unpublished data) demonstrate that lipids play a very important role in the IC life cycle of N. caninum. Increased lipid droplets’ formation and association with the parasitophorous vacuole has been demonstrated in infections by other parasites including T. gondii [22,23], Trypanosoma cruzi [21,24,25], Leishmania amazonensis [26], Plasmodium falciparum [27], and P. berghei [28]. However, to date the mechanisms that govern lipid droplets’ biogenesis and its role to N. caninum pathogenesis are not known.Further, we determined the temporal changes in exometabolome composition by obtaining Raman spectra from medium of infected and control cultures. PCA scores plots and loadings plots showed a clear separation between samples taken from infected and control cultures (Figure 5), supporting the feasibility of this method for the investigation of the biochemical differences between control and infected cultures. These data also indicate that footrpinting metabolic analysis using label-free Raman spectroscopic imaging combined with multivariate chemometric analysis has enough resolution to monitor infection-related metabolic changes over the course of time spanning the IC life cycle of the parasite. This time-resolved-based analysis is essential since metabolic differences can be highly dependent on growth phase of the cell, and cellular biochemistry changes during growth of both the cell and the parasite.

The detection of signals for nucleic acids (1046 cm^−1^ and 1420 cm^−1^) in culture medium of control and infected cells was interesting (Figure 6). The source of the nucleic acids’ traces, host or parasite origin, is unknown. However, the concentration of nucleic acids was found to increase in the medium of infected cultures in proportion to the proliferation of parasite, suggesting the source of the nucleic acids to be of parasite origin. Apoptosis and/or necrosis are the two main mechanisms involved in release of DNA from normal or diseased living cells. However, parasite-infected cells are known to resist apoptosis [12,29], countering the notion of apoptosis as the main mechanism for generating free DNA. The increase in nucleic acid signals in infected culture at 18 h PI followed by a decrease at 24 h PI (Figure 6) correlates with the measurement of the intracellular LDH enzyme release, where N. caninum was found to compromise the membrane integrity of infected cells in the first 18 h PI (Figure 2), increasing the nucleic acids’ permeability. Interestingly, the nucleic acids Raman signals peak again at 48 hr PI around the time the parasite is about to exit the cells. These findings indicate that although necrosis (and perhaps apoptosis) may contribute to the supernatant DNA, both mechanisms are the not the only source of extracellular DNA. More research is needed in order to determine the mechanism(s) of release and the significance of circulating DNA in the supernatant of both control and infected cultures.

## Conclusion

The main novelty of our study is that we characterized the metabolic response and viability of BBB endothelial cells to protozoal infection using a multidisciplinary approach. Analysis of the alterations in the biochemical composition of culture media obtained by using Raman microspectroscopy footprinting and chemometric analysis complemented data provided by standard biochemical assays. This integrated approach allowed the determination of the extracellular metabolites that are secreted and/or excreted from infected and non-infected cells into growth media. PCA scores plots showed a clear separation between metabolites from infected and control cultures. *N. caninum* challenge induced changes in energy status of infected cells and lipid composition of culture media. Levels of precursors needed for lipid biosynthesis increased in infected HBMECs, confirming the crucial role of lipid metabolism in the membrane biogenesis of new parasite particles. Differences detected by Raman imaging were attributed to variations in content of lipids and nucleic acids in infected cultures. At this moment, we do not know which biosynthetic step is critical for producing these changes in infected cells. We expect this and other questions to be answered in future experiments.

## Abbreviations

ATP: Adenosine 5′-triphosphate; BBB: Blood brain barrier; BHB: β-hydroxybutyrate; CNS: Central nervous system; ELISA: Enzyme-linked immunosorbent assay; HBMECs: Human brain microvascular endothelial cells; HDL: High-density lipoprotein; IC: Intracellular; LDH: Lactate dehydrogenase; LDL: Low-density lipoprotein; MOI: Multiplicity of infection; NEFA: Non-esterified (unsaturated) fatty acids; PBS: Phosphate-buffered saline; PCA: Principal component analysis; RPMI: Roswell Park Memorial Institute medium; PI: Post infection; TGA: Triglycerides.

## Competing interests

The authors declare that they have no competing interests associated with the publication of this manuscript.

## Authors’ contributions

HME conceived and designed the experiments. HME and MMA performed the experiments and wrote the initial draft of the manuscript. KK assisted with Raman Spectroscopy analysis. ZXQ provided intellectual advice during the planning and execution of the experiments. All authors read, revised and approved the final manuscript.
